# Method for Quantitative Study of Airway Functional Microanatomy Using Micro-Optical Coherence Tomography

**DOI:** 10.1371/journal.pone.0054473

**Published:** 2013-01-23

**Authors:** Linbo Liu, Kengyeh K. Chu, Grace H. Houser, Bradford J. Diephuis, Yao Li, Eric J. Wilsterman, Suresh Shastry, Gregory Dierksen, Susan E. Birket, Marina Mazur, Suzanne Byan-Parker, William E. Grizzle, Eric J. Sorscher, Steven M. Rowe, Guillermo J. Tearney

**Affiliations:** 1 Wellman Center for Photomedicine, Harvard Medical School, Massachusetts General Hospital, Boston, Massachusetts, United States of America; 2 Department of Dermatology, Massachusetts General Hospital, Boston, Massachusetts, United States of America; 3 Department of Pediatrics, University of Alabama at Birmingham, Birmingham, Alabama, United States of America; 4 Harvard-MIT Division of Health Sciences and Technology, Cambridge, Massachusetts, United States of America; 5 Harvard Medical School, Boston, Massachusetts, United States of America; 6 Cystic Fibrosis Research Center, University of Alabama at Birmingham, Birmingham, Alabama, United States of America; 7 Department of Medicine, University of Alabama at Birmingham, Birmingham, Alabama, United States of America; 8 Department of Pathology, University of Alabama at Birmingham, Birmingham, Alabama, United States of America; 9 Department of Pathology, Massachusetts General Hospital, Boston, Massachusetts, United States of America; University of Pittsburgh, United States of America

## Abstract

We demonstrate the use of a high resolution form of optical coherence tomography, termed micro-OCT (μOCT), for investigating the functional microanatomy of airway epithelia. μOCT captures several key parameters governing the function of the airway surface (airway surface liquid depth, periciliary liquid depth, ciliary function including beat frequency, and mucociliary transport rate) from the same series of images and without exogenous particles or labels, enabling non-invasive study of dynamic phenomena. Additionally, the high resolution of μOCT reveals distinguishable phases of the ciliary stroke pattern and glandular extrusion. Images and functional measurements from primary human bronchial epithelial cell cultures and excised tissue are presented and compared with measurements using existing gold standard methods. Active secretion from mucus glands in tissue, a key parameter of epithelial function, was also observed and quantified.

## Introduction

Mucociliary transport and the function of the airway surface is an area of active study of the human respiratory system. In healthy airways, a layer of cilia continuously transports airway mucus, a vital mechanism for defense against particulate contamination and biological invaders. In many respiratory diseases, however, this mechanism weakens or fails. Perhaps the best known of these is cystic fibrosis (CF) airway disease, in which a mutation in the cystic fibrosis transmembrane conductance regulator (CFTR) impairs the clearance of mucus from the lungs and airways [1], [2], [3]. Chronic obstructive pulmonary disease (COPD) also causes compromised mucus flow [4], as does primary ciliary dyskinesia, each through distinct mechanisms.

To investigate the pathogenesis, progression, or treatment of these diseases, a tool to quantitatively characterize the functional microanatomy of living cells and tissues without disturbing the mucociliary mechanism is highly desirable. Relevant metrics include the airway surface liquid (ASL) depth, the thickness of the thin layer of liquid surrounding the cilia known as the periciliary liquid (PCL) depth, the ciliary beat frequency (CBF), and the velocity of mucociliary transport (MCT).

Although individual methods exist for the measurement of ASL, PCL, CBF, and MCT, each has significant limitations. ASL can be measured with X–Z scanning confocal microscopy, but requires transient dyes [5], [6] and is not readily performed *in vivo*. PCL measurements require osmium tetroxide fixation with perfluorocarbon preservation of the ASL, a technique that is destructive and cannot be performed on living tissues. Particles and radionuclide labeling can be used to measure the rate of MCT *in vitro* and *in vivo*, respectively [7], [8], but the addition of exogenous material may perturb the mucus transport itself, causing unreliable measurement [8], and radionuclear studies do not provide cellular level detail. High frame rate phase contrast microscopy can be used to measure CBF [9], [10], [11], but can only be used in trans-illumination geometries and therefore have limited *in vivo* potential. Furthermore, each of these individual techniques involves separate imaging equipment and contrast mechanisms, a prohibitive impediment to the comprehensive dynamic study of mucociliary transport, including the inter-relationships between these parameters, which requires the acquisition of each of these measurements simultaneously.

Optical coherence tomography (OCT) [12], [13] produces cross-sectional images based on sample reflectance, which is well suited for the study of airway microanatomy, since a cross-sectional view would reveal the thicknesses of the ASL and PCL as well as capture tissue structure simultaneously. Moreover, since imaging can be conducted non-invasively and at video rate, functional parameters can be derived without perturbing the airway surface. Contrast is derived from the natural reflectance of the various liquid and cell layers without the need for exogenous dyes, which enables ASL, PCL, CBF, and MCT measurements simultaneously from the same set of images. However, though macroscopic measurements such as mucus transport rate or thick ASL depths can be made with relatively low resolutions [14], most OCT systems do not possess sufficient resolution to fully resolve ASL and PCL layers in normal and CF airway epithelia [15], [16], [17], [18], and the ciliary layer, a requirement for accurate measurement of CBF. ASL and PCL thicknesses are critical for maintaining normal mucociliary clearance, since an ASL depth decrease as small as a few microns may result in failure of the mucociliary apparatus in HBE cultures [17], and increases as small as a couple of microns can contribute to restoration of CF airway epithelial function [19], [20]. Since cilia provide the primary driving force for mucociliary clearance, the ability to resolve individual cilia and directly measure their activity including CBF and ciliary beat pattern is of paramount importance.

The resolution of an OCT system intended for mucociliary investigation should ideally be smaller than the height difference between the effective stroke and recovery stroke (∼2 µm) [17], [21] in order to investigate the ciliary motion pattern within the PCL space. The stroke pattern of motile cilia [21], which entails a complex cycle of recovery, effective forward motion, and rest, could be revealed with sub-ciliary resolution, which would open to study an entirely new set of previously inaccessible functional parameters. A recent publication has indicated that the presence of ciliary beating can be detected even with an axial resolution of approximately 3 µm, but a direct quantitative measure of CBF could not be made, nor was the ciliary stroke pattern imaged [14].

We have developed an OCT system with 1-micron resolution, a technique we have named micro-OCT (μOCT). Previously published results from human and swine coronary arteries using our μOCT system revealed unprecedented subcellular detail in these tissues [11]. In the present work, we demonstrate μOCT applied to living airway epithelium, both in cultures and in tissues *ex vivo*, including direct and simultaneous measurements of ASL, PCL, MCT, ciliary stroke pattern, CBF, and glandular function without exogenous labeling, providing a new tool to interrogate the functional microanatomy of respiratory epithelia with unequaled resolution.

## Materials and Methods

### Ethics Statement

Use of human cells and tissues was approved by the Institutional Review Boards at University of Alabama Birmingham (IRB #X080625002) and Massachusetts General Hospital (IRB #2008P000178). Primary human bronchial epithelial cells were derived from lung explants after written informed consent was obtained from non-CF subjects with confirmed CFTR genetics. Remnant human tissues following organ explantation were acquired after written informed consent was obtained from non-CF subjects with confirmed CFTR genetics. The Subcommittee for Animal Research Care at the Massachusetts General Hospital (IACUC 2011N000081 and 2010N000242) approved the use of discarded swine tissue for these studies.

**Figure 1:**
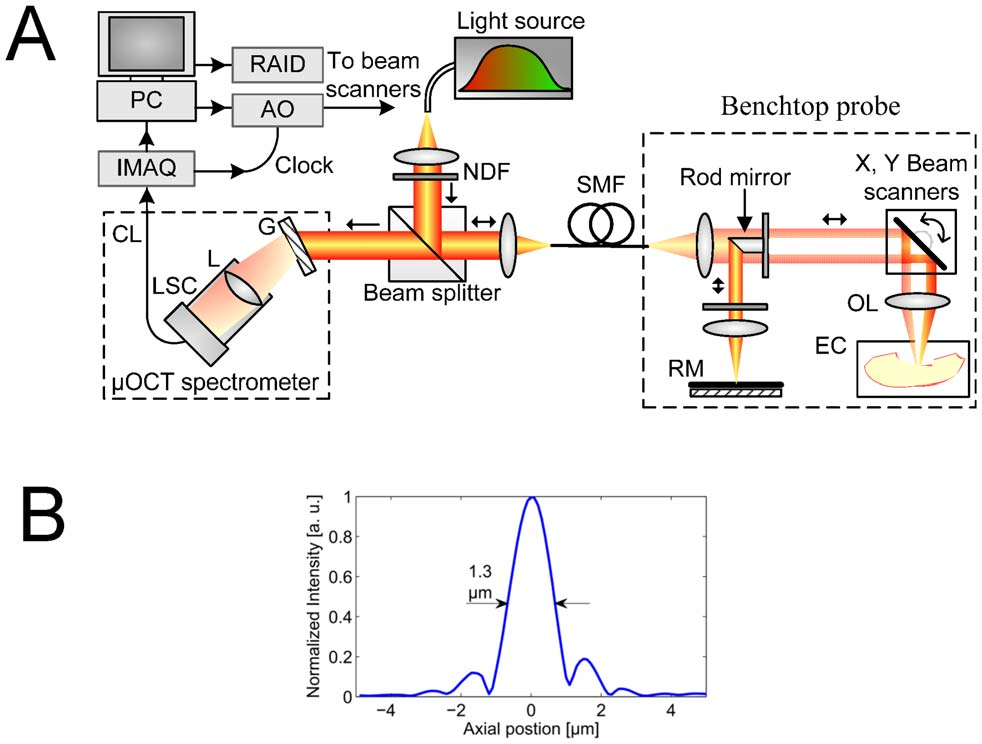
μOCT instrumentation schematic and axial resolution. A. System diagram. RM: reference mirror. OL. objective lens. EC: environmental chamber. AO: analog output board. G: grating. IMAQ: image acquisition board. L: camera lens. LSC: line scan camera. SMF: single mode fiber. PC: personal computer. RAID: redundant array of independent disks. CL: Camera Link cable. B. Depth profile of mirror surface, indicating axial full-width half maximum of 1.3 µm.

The μOCT system is a spectral-domain OCT implementation [22], [23], [24], [25] with several key improvements to standard OCT that yield high resolution in both lateral and axial directions. The general layout and axial resolution characterization are shown in Figure 1. A super-continuum source (Fianium SC450) provides the high-bandwidth, short coherence length light necessary for high axial resolution (1.3 µm, Fig. 1B). A typical OCT system includes an interferometer with the reference and sample arms intersecting at a beamsplitter. The beamsplitter is replaced in μOCT with a 45 degree rod mirror, which apodizes the sample beam by introducing a circular obscuration in the center to achieve a balance of good lateral resolution (2 µm) and long depth of focus (0.2 mm). Custom software is employed to control the galvanometer scanning motors while acquiring spectral data from the line camera. The system operates with user-configurable line and frame rates and customizable scan geometry; typical settings are 32 or 40 frames per second, 512 A-lines per frame in a linear scan, and 0.5 mm by 0.5 mm (X by Z) for a cross-sectional image. The effective thickness of each cross-section is equal to the μOCT beam spot size (2 µm).

Primary human bronchial epithelial (HBE) cells were derived from lung explants according to previously described methods [26], [27]. First or second passage cells following expansion were seeded on permeable supports for studies. At 80–90% confluency, cells were seeded onto 1.12 cm^2^, 12 mm permeable Snap-well supports (10^6^ cells per filter; Corning Inc., Corning, New York) or 6.5 mm permeable supports (0.5×10^6^ cells per filter; Corning Inc.) that were coated with NIH 3T3 fibroblast conditioned media. Cells were grown in differentiating media for at least 6–8 weeks containing DMEM/F12 (Invitrogen, Carlsbad, California), 2% Ultroser-G (Pall, New York), 2% Fetal Clone II (Hyclone, Logan, Utah), 2.5 µg/mL insulin (Sigma-Aldrich), 0.25% bovine brain extract (LONZA), 20 nM hydrocortisone (Sigma-Aldrich), 500 nM Triodothyronine (Sigma-Aldrich), 2.5 µg/mL transferrin (Invitrogen), 250 nM ethanolamine (Sigma-Aldrich), 1.5 µM epinephrine (Sigma-Aldrich), 250 nM phosphoethanolamine, and 10 nM retinoic acid (Sigma-Aldrich) until terminally differentiated.

Normal piglet tracheas were obtained from Exemplar Genetics (Sioux Center, Iowa). Tissue were dissected from one-day-old piglets and shipped on wet ice in DMEM. A modified protocol based on airway tissue handling and preparation methods developed by Ballard et al. in [28] was employed. Tracheas were immersed in 80 mL Ringer bicarbonate solution (KRB) baths at room temperature and slowly warmed to 37°C. After four hours of pretreatment, the tracheas were removed from the KRB. Accessible mucus and liquid were aspirated from the airway lumens and the tracheal ends were cannulated so that the serosal surface was bathed in KRB [29] without contacting the mucosal surface, as previously described [28], [30]. Tracheas were allowed to equilibrate in KRB bubbled with 95% O_2_ and 5% CO_2_ at 37°C and the luminal side exposed to conditioned air at 100% humidity for 2 hours prior to μOCT imaging [31].

μOCT imaging was performed on HBE cell cultures with illumination incident on the apical side of the cells. The axis of the imaging optics is typically placed within 10 degrees of normal to the cell plane to minimize errors in geometric measurements. ASL and PCL were measured directly from the thicknesses of the visible layers in the image with a correction applied for the index of refraction in the liquid (n = 1.33). ASL and PCL were evaluated at 5 equally distributed regions of the image. CBF and MCT were determined from a time series of images. CBF was measured by finding the frequency of peak amplitude in the temporal Fourier transform of the regions exhibiting oscillatory behavior. Up to 10 regions of ciliary activity per image sequence were assessed for CBF. MCT was computed by measuring the displacement of 5 to 10 visible inclusions in the mucus through time. All image analysis was performed with ImageJ and Matlab.

Comparisons to the standard optical methods for ASL, CBF, and MCT measurements were performed. For all paired comparisons, imaging was performed 1 mm from the edge of each well. For ASL depth, the HBE cell surfaces were stained with Texas Red dye (25 µL at 2 mg/mL in FC-70, administered 2 hours prior to measurements). Transwell membranes were placed in a sterile glass bottom dish coated with MEM. A confocal microscope (Carl Zeiss, Oberkochen, Germany) was used to acquire XZ cross-sectional scans. 4 regions of interest (ROI) were analyzed for each monolayer [32] and average ASL depth was measured for 5 equally distributed locations in each ROI. For CBF, cells were equilibrated at room temperature for 15 minutes, then evaluated using Hoffman contrast microscopy as described previously [33] to acquire images from 4–5 regions of interest in each well at 100 frames per second. Analysis was performed with Sisson-Avon Video Analysis (SAVA, Ammons Engineering, Mount Morris, Michigan). For MCT rate, a 50 µL suspension of 1 µm diamine polyethylene glycol (PEG) coated fluorescent beads (Molecular Probes, Eugene, Oregon) was introduced to the mucus layer. Fluorescence imaging (488 nm excitation, 519 nm emission) was performed with an inverted microscope (Nikon Diaphot, Melvin, New York). Images were analyzed using Metamorph 7.0 software (Molecular Devices, Sunnyvale, California), and transport rate was measured for 10–15 particles per region.

Excised porcine trachea tissue was also imaged with μOCT, with ASL, PCL, CBF and MCT measurements acquired with the same methods as with cultured cells. Additionally, gland ducts exhibiting mucus extrusion were imaged, and the output flow rate computed by multiplying velocity (measured in the same manner as MCT) with the cross-section of the extrusion (measured geometrically).

Finally, to demonstrate the suitability of μOCT to human tissue in addition to swine, samples of human trachea tissue were also imaged, derived from normal donor explant organs not selected for lung transplantation. Lung, mainstem bronchi, and trachea were resected en-bloc, transferred on wet ice, and large airways excised. Airway tissues were then immersed in ice cold DMEM following resection for transfer, then allowed to equilibrate to room temperature prior to μOCT imaging.

## Results

### Imaging respiratory epithelial functional anatomy in live motion

**Figure 2:**
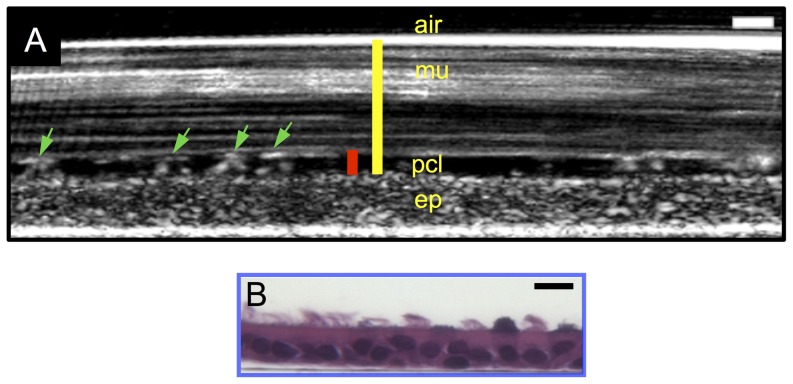
Representative μOCT image of primary HBE culture. A. A time-averaged (2 s) μOCT image of fully differentiated primary human bronchial epithelial cells derived from a normal subject. From top to bottom, air, mucus layer (mu), PCL layer (pcl; red bar), cilia (green arrows), and epithelial monolayer (ep) are readily seen. ASL depth is defined as the distance between the air-mucus interface and the apical surface of the epithelium (ep). PCL depth is defined by the distance between the ventral surface of the mucus layer and the apical surface of the epithelium. B. H&E stained histology of HBE cells. Scale bar: 10 µm.

A representative μOCT image of HBE cells (Fig. 2 A) illustrates the resolvable features of airway epithelium culture. The epithelial monolayer and the cilia can be visualized with a resolution comparable to medium power histology (Fig. 2 B). Mucus and PCL layer that together comprise the airway surface liquid (ASL) layer can also be clearly visualized (Fig. 2 A and Movie S1). From top to bottom, the air has no μOCT signal; the mucus layer appears heterogeneous with high μOCT signal intensity; the PCL gel has a low μOCT signal intensity compared with the mucus and monolayer and includes ciliary structures. The air-liquid interface, mucus-PCL interface, and apical cell surface are clearly defined so that ASL and PCL heights can be directly measured with sub-micrometer resolution. In addition to the different layers, cilia tips can be readily detected by μOCT, as they are brighter than the surrounding PCL and mucus. The tips maintain contact with the deep surface of mucus blanket and lift the mucus nearby by a few hundred nanometers during the effective stroke (Fig. 2 and Movie S1), a finding that is consistent with previous observations made using electron microscopy [21].

**Figure 3:**
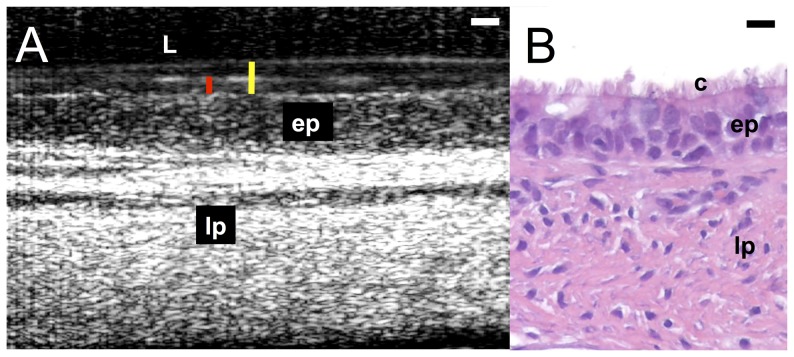
Functional anatomy of excised swine trachea. A. μOCT image. Yellow bar indicates airway surface liquid (ASL) depth and Red bar indicates PCL depth. Epithelium (ep) and lamina propria (lp) are also visible. B. H&E stained histology image illustrating cilia (c), epithelium (ep) and lamina propria (lp). Scale bar of both images: 10 µm.

Freshly excised full-thickness airway tissue retains functional mucociliary clearance under physiologic conditions. The μOCT image of fresh swine tracheal tissue (Fig. 3 A) shows cilia, epithelium and lamina propia, much as they appeared in histology (Fig. 3B). ASL and PCL layer can be clearly visualized and directly measured. A video (Movie S2) demonstrates the activity of the mucociliary apparatus. Moving mucus and beating cilia are once again clearly seen and can be readily quantified (Table 1).

**Table 1:** μOCT measured parameters from swine trachea *ex vivo*.

*Parameter*	*Value ± SEM (n = 10)*
ASL Depth	9.01±0.89 µm
PCL Depth	6.96±0.47 µm
CBF	10.55±0.12 Hz
MCT Velocity	88.9±1.8 µm/sec

Number of samples *n* refers to separate measurements from a single tissue sample.

**Figure 4:**
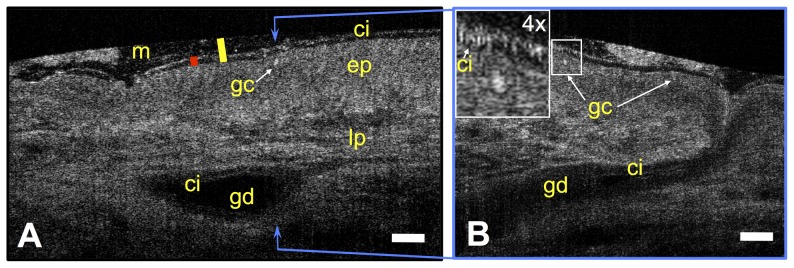
μOCT functional anatomy of human trachea. A. A time-averaged (1 s) μOCT image of human tracheal tissue shows epithelium (ep), lamina propria (lp), gland duct (gd), mucus (m), cilia (ci) and goblet cells (gc). Yellow bar indicates airway surface liquid (ASL) depth and Red bar indicates PCL depth. B. Orthogonal view at the position indicated by the dashed blue line shows the whole gland duct and the goblet cell in A. Ciliary beat pattern and possible goblet cell nucleus are clearly seen in the inset. Scale bars: 20 µm.

In addition to animal tissue, we also imaged human tracheal tissue acquired from a failed donor lung. μOCT images (Fig. 4 and Movie S3) of human tissue reveal exactly the same anatomical features as in HBE cells and animal tissues.

### Label-free, comprehensive quantification of mucociliary clearance

**Figure 5:**
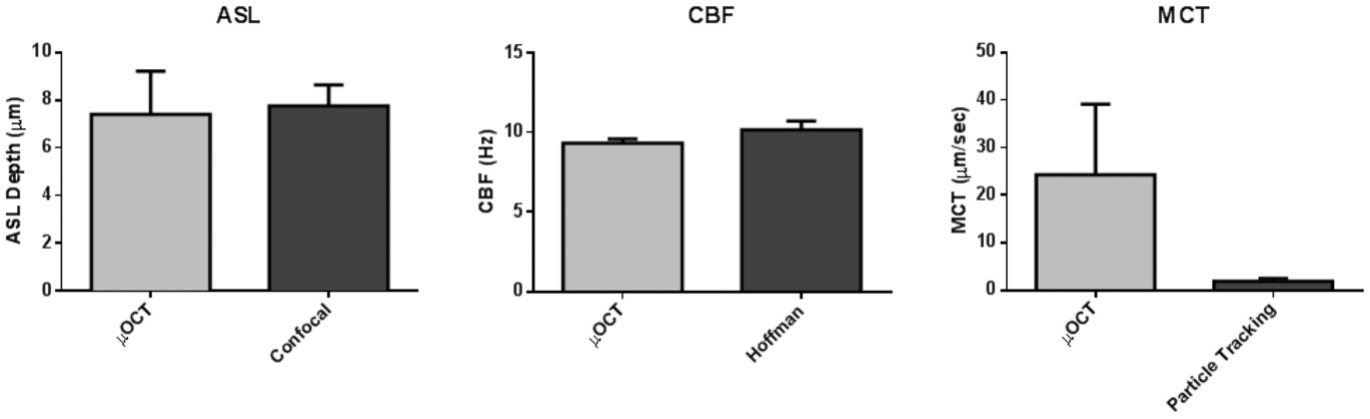
Comparison of μOCT and gold standard measurements in HBE cells. All error bars represent SEM. A. ASL depth measured with μOCT (7.40±1.82 µm, n = 5) and confocal microscopy (7.76±0.87 µm, n = 6). B. CBF measured with μOCT (9.32±0.27 Hz, n = 4) and Hoffman contrast microscopy (10.17±0.56 Hz, n = 4). C. MCT velocity measured with μOCT (24.22±14.88 µm/sec, n = 6) and particle-tracking fluorescence microscopy (1.91±0.62 µm/sec, n = 11). Number of measurements n refers to separate wells analyzed.

The high resolution and live motion capabilities of μOCT enable accurate quantification of most of important MCC metrics without aid of any exogenous contrast agent. Measurements of ASL, CBF, and MCT from μOCT imaging of HBE culture were confirmed by conventional measures of ASL depth (Fig. 5 A), CBF (Fig. 5 B), and MCT (Fig. 5 C). Notably, transport rates by particle tracking were significantly lower than those observed by μOCT, due to agglomeration of mucus around the fluorescent beads [34]. μOCT MCT rates were not affected by the phenomenon, and consequently MCT was similar to rates observed in live motion capture from intact tracheal tissue [35].

μOCT imaging was performed on porcine trachea and the same techniques previously employed to extract functional microanatomy data from HBE culture were used in tissue for the same purpose. The resulting ASL, PCL, CBF, and MCT numbers are listed in Table 1.

**Figure 6:**
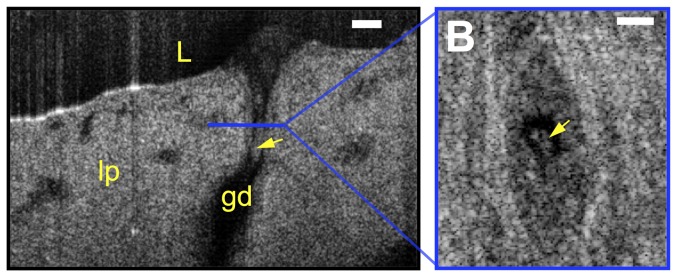
Mucus extrusion from single gland duct. A. A representative frame from a μOCT video of trachea dissected from a swine shows mucus (yellow arrow) extrusion from a gland duct (gd) in lamina propria (lp). B. Three-dimensional reconstructed en face view allows estimation of luminal area of the duct. In swine trachea, mucus extrusion rate is 0.095 nL/min (N = 3, ±SEM = ±0.006) at room temperature.

Functional data was also extracted from videos of active mucus glands in *ex vivo* swine tissue. Fig. 6A and Movie S4 shows mucus extrusion through a gland duct in 2D real-time cross-sectional images. Additionally, 3D reconstruction of the μOCT image allows estimation of the gland duct cross-sectional area in the mucus transport (Fig. 6 B), so that mucus transport rates of luminal contents can be estimated by multiplying the gland duct cross-sectional area with the longitudinal extrusion rates of mucus estimated from the real-time cross-sectional images. Calculated average extrusion rate in normal swine under room temperature is 0.095 nL/min (N = 3, ±SEM = ±0.006), similar to rates estimated from a previous study [36].

### Visualizing ciliary motion in cross-sectional view

**Figure 7:**
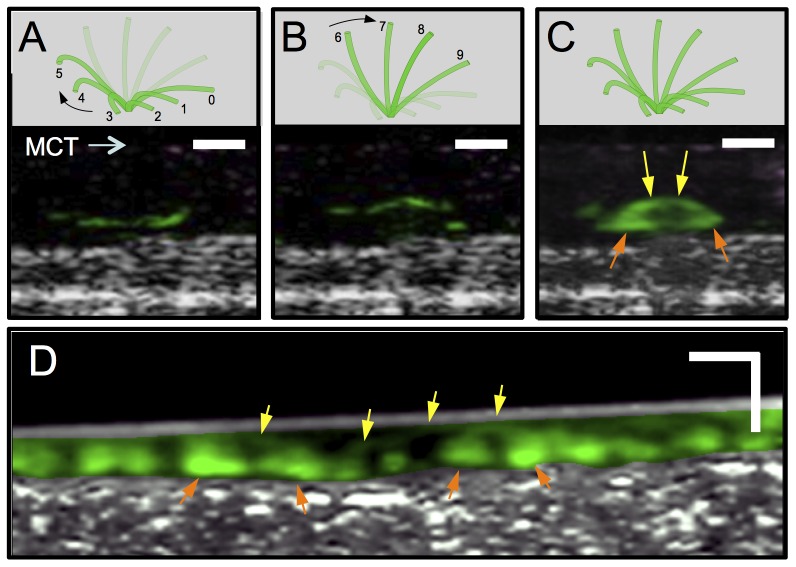
μOCT images of ciliary motion pattern in HBE culture and swine trachea. A. (Top panel) 6-stage schematic of ciliary motion during the recovery stroke; (bottom panel) a μOCT image of fully differentiated primary HBE cells derived from a normal subject shows cilia tips (green) 3–5 µm from the apical cell surface, indicating the recovery stroke. Cilia and mucus are presented in pseudo-colors: green and purple respectively. B. (top panel) 4-stage schematic of ciliary motion during the effective stroke; (bottom panel) μOCT signal of the same cilia after 250 ms that subtend an angle of 114°, delineating an arc with radius of approximately 7 µm during the effective stroke. C. (top panel) 10-stage schematic of ciliary motion during the full ciliary beat cycle; A time-averaged (4 s) image (bottom panel) shows an arc indicating the effective strokes (yellow arrows) and bilobular pattern of the recovery stroke (orange arrows). D. A time-averaged (1 s) image of normal swine trachea shows arcs indicating the effective strokes (yellow arrows) and bilobular pattern suggesting the recovery stroke (orange arrows) in the ciliary motion pattern. Scale bars: 10 µm.

**Figure 8:**
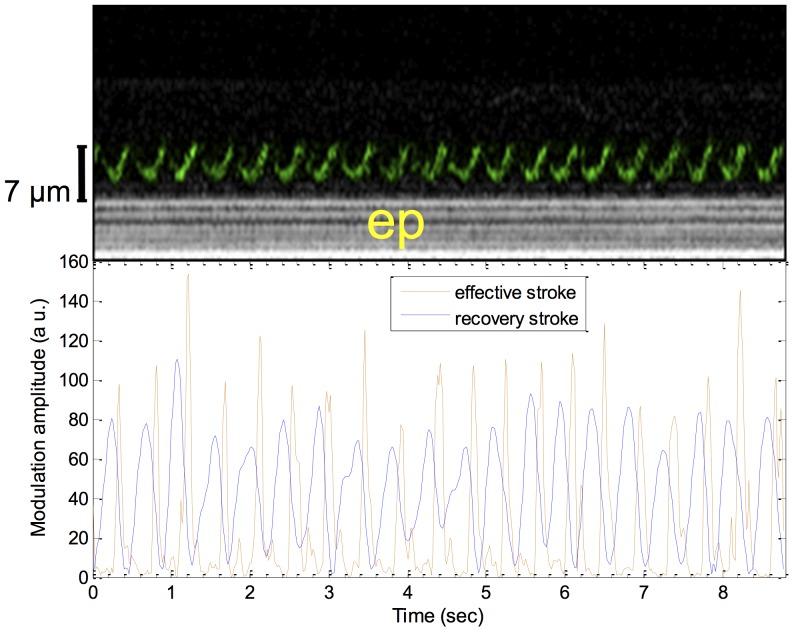
Cilia motion pattern in cultured HBE cells. Time-lapsed ciliary motion pattern can be easily identified in the M-mode image (top) of the active epithelial area shown in Figs. 7 A–C and also Movie S5. The continuity of the sinusoidal pattern in the M-mode image indicates that the entire beat cycle was captured. Corresponding time-lapse intensity analysis (bottom) reveals triphasic pattern of the ciliary beat cycle: the recovery stroke (blue line), the effective stroke (orange line) and the rest phase in between the effective stroke and next effective stroke.

The high resolution and live motion capabilities of μOCT also enable, for the first time to the best of our knowledge, visualization of ciliary motion in cross-sectional view (Fig. 7 and Movie S5). The cilia beat cycle can be divided into recovery and effective strokes, illustrated schematically in the top panels of Figs. 7A and 7B, respectively, with Fig. 7C showing the full cycle. The recovery stroke begins with the cilium in fully forward extension (position 0), then bending and rotating backwards in a clockwise sweep beneath the mucus. In the effective stroke, the cilium extends outwards towards the mucus and transcribes an approximately 110° arc in the cross-sectional plane, moving in the direction of mucus transport [21]. μOCT images provide a means to analyze the relative state of ciliary activity. In μOCT images, cilia tips appear as high intensity aggregated point scatterers. During the recovery stroke, the cilia tips appear at lower positions (3–5 µm from the apical cell surface) than in the effective stroke when they extend to their full length of ∼7 µm (Figs. 7A and 7B, lower panels). A time-averaged cross-sectional μOCT image shown in Fig. 7C demonstrates a typical ciliary beat pattern seen in μOCT images, which is characterized by an arc pattern with a peak 7 µm above the apical cell surface (yellow arrow) and a bilobular pattern 3–5 µm above the apical cell surface and just below the arc, indicating recovery strokes (orange arrow). In intact swine trachea, a similar motion pattern can also be identified (Fig. 7D). An alternative presentation for beating cilia is M-mode (Fig. 8A), where the vertical axis is depth and the horizontal axis is time. In this view, the beating cilia appear as a periodic intensity modulation. The triphasic pattern [37] of ciliary motion is shown in Fig. 8 B by decomposing the μOCT ciliary signal in Fig. 8 A into recovery/rest phase (below 5 µm) and effective phase (above 5 µm). The signal intensity and relative duration of the effective stroke likely reflect the strength of ciliary motion, potentially providing information regarding the functional microanatomy of the relative state of cilia [21].

## Discussion

The high resolution of μOCT enables the straightforward measurement of key functional parameters from airways: ASL depth, PCL depth, ciliary beating including CBF, and MCT rate, as well as extrusion rates of the submucosal airway glands. ASL and PCL depths are simple geometric measurements that can be obtained from μOCT images and can be readily discerned due to the high natural contrast in cells and tissues. To accurately and sensitively measure PCL, axial resolution must be a fraction of typical PCL thickness under pathophysiologic condition, which is approximately 7 µm in normal epithelium and ∼3 µm in cell cultures acquired from cystic fibrosis subjects [17]. The highest resolution OCT study of airways to date had 3 µm axial resolution [14], whereas μOCT achieves an axial resolution of 1 µm in tissue. Additionally, the ability to resolve length scales much smaller than the PCL itself enables the visualization of objects moving within the PCL space, such as the beating cilia or particulates within the mucus. Minute but physiologically significant changes in these parameters can thus be discerned and sensitively resolved over time and space, providing the ability to monitor functional microanatomy. With this resolution, accurate measurements of CBF can also be obtained directly from a time-series stack of B-mode (cross-sectional) μOCT images by measuring the peak frequency of oscillatory behavior, as opposed to B-mode speckle contrast techniques that do not provide quantitative measures of actual CBF [14]. The speed of μOCT will also allow rapid scanning of large segments of tissue, a quality that will facilitate *in vivo* imaging.

As a result of the high resolution of μOCT, cross-sectional ciliary stroke pattern can be distinguished in cross-sectional live imaging for the first time as observed by changes in position (Figs. 7 and 8). The entire beat cycle can be captured if the imaging plane is oriented such that the cilia tips remain within the 2 µm thickness of the cross-sectional image through the full beat (Fig. 8). Alteration in the duration of the effective stroke, the recovery stroke or the resting state can reflect response to stimulation and would be expected to confer significant changes on MCC in addition to CBF itself, in addition to altered ciliary motility. For example, a number of genes that alter ciliary stroke patterns have been identified in primary ciliary dyskinesia [38]. Other physiologic stimuli, such as transient and local perturbations of the airway surface microenvironment induced by pressure [39], [40], [41], [42] or tonicity [43] are known to alter ciliary beating, and could be perceived by μOCT for the first time as a means to analyze the relative state of ciliary activity in living cells and tissues. Given the structural equivalence of cilia in all mammalian manifestations, μOCT can also potentially be used to image any ciliated epithelia, such as ependyma and oviduct, each affected in significant and common human disease such as hydrocephalus and infertility, among others.

To validate measurements made by μOCT, comparisons were made using HBE imaging between μOCT and the gold standard techniques previously available for ASL depth, MCT rate, and CBF measurements. The ASL measurements were in very close agreement (Figure 5A). The CBFs measured with μOCT and Hoffman microscopy were also within the margin of error (Figure 5B), though we noted the Hoffman results were systematically elevated relative to the μOCT measurements from the same wells, possibly as a result of a temperature increase during prolonged microscope illumination during Hoffman imaging. MCT rate differed significantly between μOCT and the fluorescence particle tracking method (Figure 5C). However, the μOCT measurement of 24.2 µm/s is much closer to published values for bronchial mucus velocity of approximately 40 µm/s [32], [44]. We believe that the introduction of exogenous fluorescent particles artificially depressed the mucus transport rate, as evidenced by mucus bundling upon fluorescent imaging, which further highlights the μOCT advantage of label-free measurement of MCT.

In our imaging of *ex vivo* normal porcine trachea (Table 1), we found functional parameters of similar magnitude to published data. A previous study of porcine trachea reported a CBF of 12.6±2.4 Hz and mucus velocity of 42±8 µm/s [7]. Measured PCL depth is also consistent with the typical 7-µm height for normal airways [17]. ASL depth in tissue is highly dependent on sample conditions and timing, but the μOCT-derived result appears reasonable given similar validated measurements from HBE cells.

Measurement of the output flow rate of mucus glands was achieved with μOCT and is another novel capability unique to this technique. Since glandular function is a key constituent of the function anatomy of the airway surface, is known to be perturbed in CF airway tissues [45], [46] and is responsive to physiologic stimuli [47], the ability to measure glandular function in situ, without microdissection, is a significant advance that could reveal new insights into airway disease pathogenesis and response to therapeutics.

The availability of a single imaging technology that can unify the measurement of many functional parameters of the airway epithelial surface opens the door for many research applications. Any study of the response of the mucociliary transport apparatus to a given condition or stimulus can now employ μOCT imaging to measure these key metrics. For example, the effect of treatments meant to restore mucus clearance in diseases with mucociliary impairment can be rapidly measured with μOCT. Imaging of the ciliary stroke pattern may also facilitate basic science research on the micro-biomechanics of mucus clearance. Because the technique is rapid and non-invasive, μOCT could also be suitable for cell-based screening of candidate pharmacologic agents that modulate the functional airway microanatomy.

μOCT has high potential for translation to *in vivo* airway imaging, both in animals and humans. Imaging results from human tissue (Fig. 4) also illustrate that μOCT can be extended from culture or animal models to intact human organs. Our ability to study tissue *in situ* is limited only by physical access. The interferometer optics of our present instrument can be replaced by a fiber probe that can be inserted into an airway lumen, facilitating endobronchial acquisition guided by fiber optic bronchoscopy, as previously described for conventional OCT imaging [48]. Such an advance could provide a crucial step forward in our understanding of the cellular mechanisms of mucociliary clearance and the response to experimental therapeutics.

## Conclusion

We have developed μOCT, a high-resolution spectral domain OCT imaging technique, and established methods and algorithms to apply μOCT to quantitatively study functional microanatomy of airways cells and tissue. The high resolution of μOCT allows many functional parameters to be measured simultaneously and directly, enabling comprehensive study of the mucociliary clearance apparatus. Of note, μOCT provides the live visualization of the phases of ciliary strokes, which is not achievable by any other current method, and readily discerns the PCL depth. Comparisons with measurements from existing techniques and known values from the literature have validated μOCT as a quantitative tool that is well suited for further *in vitro* and *ex vivo* investigation, cell-based functional screening and ultimately, translation for human use *in vivo*. Our future work will employ the imaging system and methods described in this article to compare CF vs. non-CF phenotypic characteristics and investigate functional responses to pharmacologic stimulation.

## Supporting Information

Movie S1 **Representative μOCT movie of primary HBE culture mucociliary transport.** A real-time movie of cultured HBE cells from a normal donor. See Figure 2 for detailed explanation of anatomy. Mucus is flowing from right to left above a layer of beating cilia protruding from the epithelium. From this image series, MCT rate can be computed by measuring the displacement of the visible inclusions in the mucus layer through time, and CBF can be measured by finding the peak frequency of the temporal Fourier spectrum of the oscillating cilia. ASL and PCL depth can also be computed from individual frames or from several averaged frames.
(AVI)

Movie S2 **μOCT video of excised porcine tissue with active mucociliary transport.** A thin layer of mucus can be seen transported from left to right by cilia. Anatomic layers labeled in representative frame. From top: lumen (L), mucus (mu), cilia and periciliary layer (PCL), epithelium (ep), and lamina propria (lp). Scale bar: 10 µm.
(AVI)

Movie S3 **μOCT video of human tracheal tissue from failed donor lung.**
(AVI)

Movie S4 **μOCT video of mucus extrusion from single gland duct in swine trachea tissue at room temperature.**
(AVI)

Movie S5 **μOCT video of ciliary motion.** Cilia and mucus are presented in pseudo-colors: green and purple respectively. The ciliary pattern is clockwise as the mucus is moving left-to-right. Scale bar: 10 µm.
(AVI)
